# Formation Mechanism and Dielectric Properties of Ultra-High-Voltage Anodic Al Foils Investigated by ReaxFF-MD and DFT

**DOI:** 10.3390/ma19112373

**Published:** 2026-06-03

**Authors:** Xuliang Chu, Yucheng Ji, Chenyang Yao, Jinlong Wu, Xiaoxue Song, Xiaoou Liu, Hongliang Li, Xin Wang, Wenfeng Yang, Junsheng Wu, Chaofang Dong

**Affiliations:** 1 National Materials Corrosion and Protection Data Center, Institute for Advanced Materials and Technology, University of Science and Technology Beijing, Beijing 100083, China; 2 Xinjiang Joinworld Co., Ltd., Urumqi 830013, China

**Keywords:** ultra-high-voltage anodized foil, dielectric film, molecular dynamics simulation, first-principles calculation

## Abstract

Understanding the atomic-scale formation of anodic oxide films is critical for Al electrolytic capacitors. The formation mechanism and dielectric properties of an ultra-high-voltage anodized Al foil were investigated by combining experiments, reactive molecular dynamics, and first-principles calculations. Results indicate that film growth is primarily governed by the (011) crystal plane, and the density of the oxide film increases with the applied electric field. The diffusion barrier of H atoms (0.54 eV) is significantly lower than that of O atoms (1.41 eV), suggesting the preferential formation of Al hydroxide oxide species on the surface. As the anodization voltage increases, the oxide undergoes phase evolution from AlOOH to γ-Al_2_O_3_ and finally to α-Al_2_O_3_. First-principles calculations reveal the dielectric constants of alumina, which are 7.96 for AlOOH, 21.80 for γ-Al_2_O_3_ (Paglia structure), and 10.24 for α-Al_2_O_3_. These findings provide a theoretical basis for optimizing the microstructure and dielectric performance of ultra-high-voltage anodized Al foils.

## 1. Introduction

**Figure 1:**
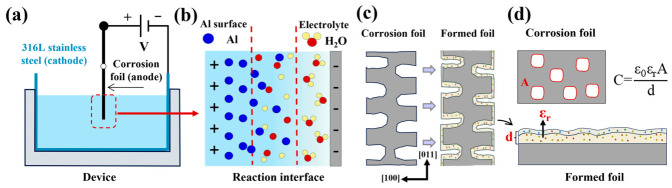
Schematic illustration of the anodic oxidation process. (**a**) Schematic diagram of the experimental setup, (**b**) Interfacial reactions during the anodization process, (**c**) Film formation process, (**d**) Factors affecting the capacitance.

Al electrolytic capacitors are essential components in modern electronic devices, which are widely used in automotive electronics and 5G communications due to their high capacitance, low cost, and ease of manufacturing [1,2,3,4]. The Al oxide film on the Al foil after anodization acts as a dielectric, defining the capacitance and breakdown of electrolytic capacitors [5,6]. These capacitor performances are collaboratively determined by the corrosion and anodization processes of Al foil, especially for the specific capacitance (C) [7]. Figure 1 summarizes the microscopic reaction process of the anode Al foil, where *C* can be calculated by the vacuum dielectric constant (*ε*_0_), the relative dielectric constant of the dielectric (*ε*_r_), the true surface area of the electrode (A), and the distance between the electrodes (d). For the corrosion process, the pores on the Al foil determined by the A are close to the physical limit [8]. Therefore, developing novel Al oxide with high C is a critical topic for the advancement of high-performance electrolytic capacitors. While the most effective ways to improve the volumetric capacitance of the anodized Al foil (Figure 1d) are increasing its *ε*_r_ and reducing its d [9,10], this remains challenging in practice.

Following corrosion, the anodization process forms the oxide film on the surface of the Al foil. For the preparation of ultra-high-voltage anodized Al foil, hydration treatment and multi-step anodization processes are commonly used. Since the dielectric constant is an intrinsic property of the crystal structure, its adjustment must be achieved by altering the microcrystalline structure of the oxide film [11,12].

To improve the performance of the anodized Al foil, various modification strategies have been reported [13]. For instance, Ban et al. [14] studied the effect of hydration treatment on Al foil anodized at 530 V and found that the crystallinity of the surface film was significantly enhanced with prolonged hydration time, thereby increasing the specific capacitance from 0.5726 μF·cm^−2^ to 0.6784 μF·cm^−2^. Dong et al. [15] used phosphoric acid and organic acid to replace boric acid at the low-voltage stage to prepare 520 V anodized Al foil. They found that organic acid could improve the withstand voltage and specific capacitance, while phosphoric acid could shorten the voltage-rising time; however, both increased the leakage current of the dielectric film. Using azelaic acid as the anodizing solution, Pan et al. [16] found that the reduced thickness of the surface oxide film promoted the formation of crystalline oxides in the 550 V anodized foil, thereby enhancing the specific capacitance. However, this leads to a decrease in withstand voltage and an increase in leakage current. Du et al. [17] coated etched Al foil with a barium titanate (BT) thin film under low-voltage conditions to serve as a partial dielectric for Al electrolytic capacitors. After annealing, the specific capacitance was found to be 46% higher than that of the uncoated foil.

For the preparation of ultra-high-voltage Al foils (≥800 V), relevant studies are limited [17,18,19]. At such extreme voltages, conventional single-step anodization cannot dynamically adjust parameters, often triggering severe local sparking and dielectric breakdown. In contrast, multi-stage formation overcomes this by enabling stage-by-stage electrolyte regulation to ensure the steady growth of high-quality oxide films. Furthermore, although doping with high-dielectric materials can enhance capacitance, it typically prolongs processing cycles and degrades the withstand voltage. These approaches are typically associated with extended processing cycles, whereas the applied voltage, leakage current, and dielectric loss performance of Al foil are inferior to those of pure anodic Al oxide films. In addition, the self-healing ability of the Al oxide film no longer exists. To break through the 800–1200 V voltage barrier in capacitor technology, it is critical to unravel the atomic-scale evolution of the oxide film and its resulting dielectric properties. Therefore, the preparation of ultra-high-voltage anodized foils and understanding the intrinsic mechanisms of the anodization reaction have become important topics for the future development of Al electrolytic capacitors.

During anodization, the pore-formatted Al foil serves as the anode, while the cathode is made of 316L stainless steel (Figure 1a). A constant voltage is applied to the Al foil, causing the dissolution of Al atoms and the formation of an oxide film on its surface (Figure 1b,c) [20,21]. At present, density functional theory (DFT) and reactive force field molecular dynamics (ReaxFF-MD) calculations have been widely used to investigate atomic-scale reaction mechanisms. For instance, Wu et al. [22] used ReaxFF-MD simulations to investigate the hydrolysis process of Al in H_2_O vapor under different electric and temperature fields, providing important guidance for laboratory hydrogen production from a computational perspective. Luo et al. [23] used DFT and MD methods to investigate the impact of various organic acids on the corrosion pore formation of Al foil. Systematic computational studies on the anodization mechanisms and dielectric evolution of Al foil are still scarce. Using computational methods to understand the chemical reactions on the surface of Al foil and the dielectric properties of the oxide film could significantly reduce the time required for systematically studying the impact of the anodization process on dielectric performance and film structure.

In this study, 800 V ultra-high-voltage anodized Al foils were prepared, and the structural evolution of the oxide film was investigated. To further understand the formation mechanism and dielectric behavior of Al oxide film, ReaxFF-MD [24] simulations were used. The reactions between different crystal faces of Al and water under varying electric field conditions were discussed. Additionally, DFT calculations were employed to investigate the diffusion of H and O atoms across the Al surface. By calculating the corresponding activation energy barriers, the preferential formation pathways of hydroxide and Al oxide were clarified. Furthermore, based on DFT calculations, the electronic and ionic contributions to the dielectric response under different external field frequencies were analyzed [25]. This combined experimental and multi-scale computations elucidates the growth mechanisms of Al oxide films and their dielectric properties during the formation of Al foils, which can provide a crucial theoretical basis for the anodization of the Al foil.

## 2. Methods

### 2.1. Material Preparation

The Al foil used in this study has a thickness of 140 μm and a purity of 99.98 wt.%. More than 97% of the surface was oriented along the (100) crystallographic plane. After electrochemical etching, a high-density tunnel pore structure aligned perpendicular to the foil surface was obtained. The etched Al foil was rinsed with deionized water and air-dried to remove surface contaminants. Subsequently, the foil was boiled in deionized water for 15 min prior to the anodization treatment.

**Figure 2:**
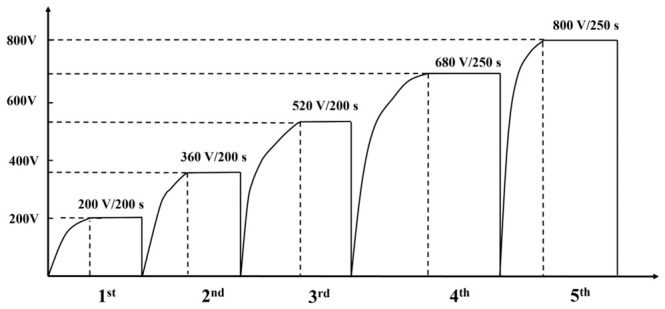
Multi-step anodization process curve.

In multi-step anodization, a 50 g·L^−1^ boric acid solution was used as the anodizing electrolyte, and ammonium pentaborate was added to enhance the conductivity of the electrolyte. The anodization process is illustrated in Figure 2. During the formation process, the temperature was maintained at 90 ± 2 °C. After a five-step anodization treatment, the final anodized Al foil exhibited a formation voltage of 800 V.

### 2.2. Characterization

To observe the oxide film growth inside the pores, the anodized foil was cleaned with deionized water to remove residual anodizing solution from the surface and then dried. Subsequently, ion beams were used to remove the surface oxide film to observe the morphology of the tunnel pores. The accelerating voltage of the ion beams was set to 7 kV, and the incident angle was maintained at 15°. The thinning time is shown in Table 1. The surface morphology of the tunnel pores was observed using a field emission scanning electron microscope (FE-SEM) at 20 kV. The tunnel pores and oxide film thickness were analyzed using ImageJ 1.53a. Additionally, energy dispersive X-ray spectroscopy (EDS) line scanning was conducted to analyze the distribution of Al and O elements within the oxide layer in the tunnel pore region.

To obtain anodic oxide films for structure analysis, the anodized Al foils were immersed in a 10 wt.% iodine–methanol solution for 12 h to remove the underlying Al substrate. The separated oxide layers were collected, repeatedly rinsed with deionized water, dried, and ground into fine powder. XRD measurements were carried out using Cu Kα radiation. The diffraction patterns were recorded over a 2*θ* range of 10–90° at a slow scanning rate of 2° min^−1^. Phase identification was performed based on the obtained diffraction data to determine the composition of the anodic oxide films.

### 2.3. ReaxFF-MD Simulation

The Al-H_2_O ReaxFF-MD simulations were performed using LAMMPS 2023 [26]. The force field parameters used in this study were derived from the Cu-Si-Al-O-H ReaxFF developed by Psofogiannakis et al. [27]. These parameters were optimized based on DFT calculations and experimental data, enabling accurate simulation of bond formation and dissociation processes in the Al-H_2_O system under different temperature and electric field conditions. The detailed mathematical expression of the ReaxFF energy function and the description of each energy term are provided in the Supporting Information (Equation (S1)) [28].

**Figure 3:**
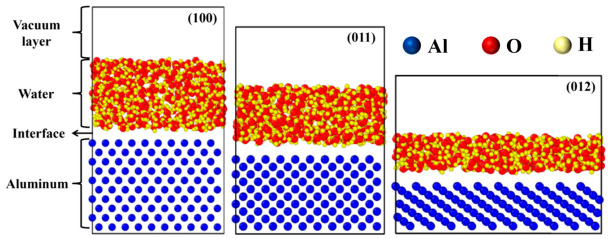
The Al-H_2_O models with different orientations in the ReaxFF-MD simulation.

Given that the (100) crystal plane constitutes 97% of the Al foil, the tunnel pore structure formed after corrosion and pore formation is shown in Figure 1c. During the multi-step anodization, different orientations of the Al foil interact with water under the influence of the electric field. Therefore, three Al-H_2_O models with the (100), (110), and (012) were constructed (Figure 3). The Al(100)-H_2_O model consists of 1000 Al atoms and 400 H_2_O molecules, with a model size of 28.6 × 28.6 × 49.2 Å^3^. The Al(011)-H_2_O model consists of 1000 Al atoms and 400 H_2_O molecules, whose size is 40.5 × 28.6 × 39.8 Å^3^. The Al(012)-H_2_O model consists of 1000 Al atoms and 400 water molecules, and its size is 49.6 × 39.6 × 30.3 Å^3^.

A 10-Å-vacuum layer was introduced along the *z*-direction for all MD models. The Berendsen thermostat was used to study the effects of temperature (363 K) on the oxidation and corrosion processes [29]. All simulations were performed in the NVT ensemble with a time step of 0.1 fs [30]. To maintain charge balance at each computational step, a strict charge equilibration method was applied. Periodic boundary conditions were set in the *x*- and *y*-directions, with a molecular reflection wall added along the *z*-direction. The atomic trajectories of all atoms in the system were output every 1000 steps.

### 2.4. DFT Calculations

DFT calculations were performed using the Vienna Ab initio Simulation Package (VASP 6.4) [31]. To simulate the H/O diffusion and surface adsorption, Al(011) surface models containing H or O atoms were constructed. These models consist of seven layers of Al atoms, with a H atom or an O atom placed on the surface. The Brillouin zone was sampled using a 3 × 4 × 1 Monkhorst-Pack grid, which was automatically determined by enforcing a uniform reciprocal space K-spacing of 0.19 Å^−1^ to guarantee integration accuracy. The positions of the bottom four layers of Al atoms were fixed to simulate bulk phase boundary conditions. During structural optimization, the top three layers of Al atoms and the adsorbates were fully relaxed. The climbing image nudged elastic band (CI-NEB) method [32,33] was used to calculate transition state energies. The minimum energy pathways were mapped using 3 intermediate images with a spring constant of 5 eV/Å^2^, and structures were optimized until the maximum force fell below 0.01 eV/Å. Furthermore, to determine the effect of pre-penetrated H atoms in the Al matrix on the subsequent surface adsorption of O atoms, the surface adsorption energy (*E_ads_*) was calculated, with the detailed governing equation and reference states described in the Supporting Information (Equation (S2)) [34].

Here, isolated atomic oxygen was chosen as the reference to strictly align with the interfacial mechanism (Equation (1)), where active oxygen species directly participate in lattice formation rather than intact molecules. Moreover, since our core analysis focuses on the relative energy difference between H-free and H-containing surfaces, the reference energy mathematically cancels out, ensuring the comparative trends remain strictly valid.

(1) $$2Al^{3+}+3O^{2-}\to Al_{2}O_{3}$$

For dielectric property calculations, DFT models were sourced from the Materials Project databases [35,36] and complemented by validated structures from the literature [37]. The evaluation of dielectric properties followed a two-step procedure: first, the electronic dielectric tensor was derived via the independent-particle approximation by calculating the optical transition matrix elements; subsequently, the ionic (lattice) contribution to the static dielectric tensor was rigorously determined using density functional perturbation theory (DFPT) [38,39,40]. Specifically, the Brillouin zone was sampled using a uniform K-point mesh with a dense spacing of 0.13–0.19 Å^−1^, complemented by a 0.01 eV Gaussian smearing. Furthermore, the self-consistent field iterations were performed with a stringent energy convergence threshold of 1 × 10^−8^ eV. Based on the optimized electronic structure, the total static dielectric constant, incorporating both electronic (*ε_ele_*) and ionic (*ε_ion_*) polarization contributions, was rigorously evaluated using the expression detailed in the Supporting Information (Equation (S3)).

## 3. Results and Discussion

### 3.1. Effect of Electric Field on Al-H_2_O Reaction

**Figure 4:**
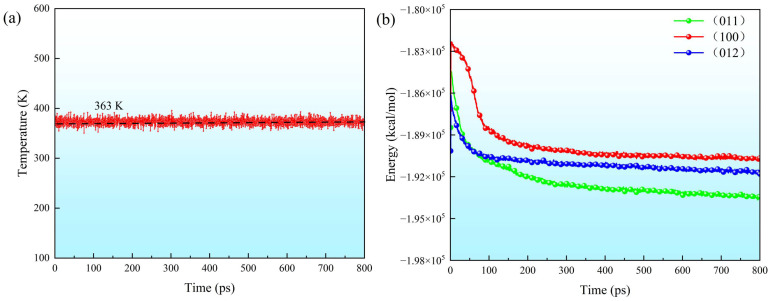
Temperature and energy variations in the Al-H_2_O system: (**a**) Temperature variation during Al-H_2_O reaction, (**b**) Total energy variation at 363K during the reaction.

To investigate the effect of the electric field on the Al-H_2_O reaction and ensure that the reaction proceeds sufficiently, Figure 4a summarizes the temperature fluctuations of the system during the simulation. It can be found that the temperature remains stable at approximately 363 K, which indicates that the system has achieved thermal equilibrium, thus enabling subsequent energy analysis. Figure 4b displays the total energy variation during the Al-H_2_O reaction process. In the first 50 ps, the total energy profiles reveal facet-dependent reaction kinetics: the (012) plane experiences the most dramatic energy dissipation, followed by the (011) plane, while the (100) plane exhibits the most gradual decline. This phenomenon is fundamentally governed by the surface morphology; surfaces with higher Miller indices typically feature smaller interplanar spacings and looser atomic configurations.

H_2_O molecules can more easily infiltrate and interact vigorously with the under-coordinated Al atoms on the (012) and (011) surfaces. Such facilitated penetration and strong interfacial bonding expedite the exothermic reaction, directly leading to the sharply steeper plunge in total energy observed for the higher-index facets. At 200 ps, the total energies of the Al(100), (011), and (012) crystal faces converge to approximately −189,873.07 kcal·mol^−1^, −192,026.67 kcal·mol^−1^, and −190,849.86 kcal·mol^−1^, respectively. Beyond this point, the absence of any further drastic energy decline indicates that the system has reached equilibrium and is ready for sampling.

**Figure 5:**
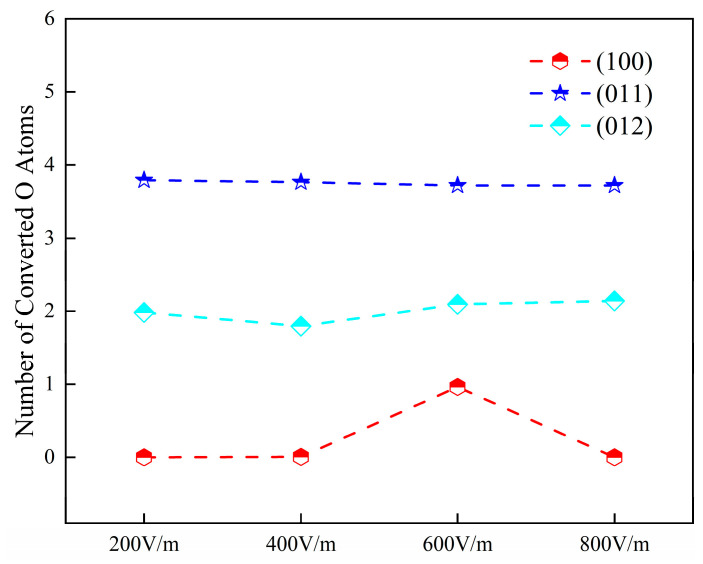
Number of transformed O atoms in Al_2_O_3_ on different crystal orientations.

During this sampling phase, the progress of the Al-H_2_O reaction was monitored by tracking the charge evolution of the O atoms. As documented in previous literature, the maximum charge of the O ions in the unreacted water is −0.99 e, while the average charge of the O ions in Al oxide is −1.25 e, with the maximum charge reaching −1.42 e. According to the charge transfer, the number of O atoms with a charge less than −1.00 e in varying crystal faces under different electric field conditions was recorded over a stable period of 500 ps (Figure 5). It is observed that the number of O ion transformations differs across the crystal faces. For (100) orientation, only a few O ions undergo transformation, and their number tends to zero. On the (012) crystal face, the number of O ion transformations fluctuates between 1.7 and 2.2, while the (011) crystal face has the most O ion transformations. Under different electric field strengths, the average number of transformations on the (011) face ranges from 3.7 to 3.8, indicating that the anodization of the Al foil is mainly dominated by the (011) orientation.

**Figure 6:**
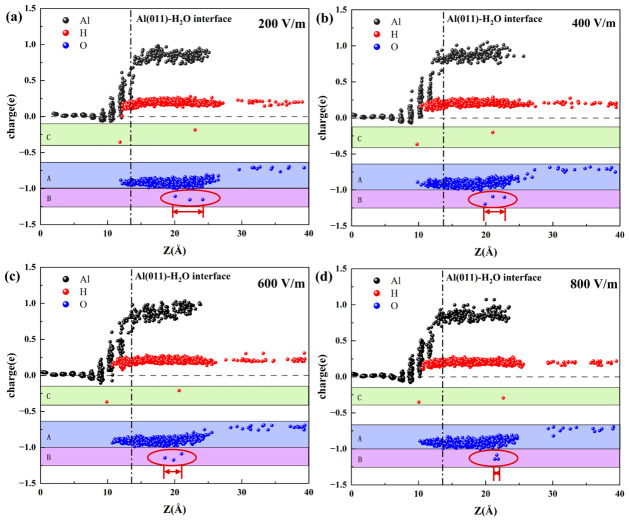
Atomic charge distribution of the Al-H_2_O system under different electric field conditions at 363K: (**a**) 200 V/m, (**b**) 400 V/m, (**c**) 600 V/m, (**d**) 800 V/m.

Furthermore, the charge distribution for the (011) crystal face is plotted in Figure 6. Region A represents the charge distribution of O atoms in unreacted water (−1.00 e < charge < −0.60 e), Region B corresponds to the O atoms incorporated into the Al oxide (−1.25 e charge < −1.00 e), and Region C denotes the charge state of the reacted H atoms (−0.45 e < charge < −0.15 e). It can be seen that a pronounced charge exchange occurs at the metal–solution interface. The metallic Al atoms lose electrons, resulting in a substantial increase in their positive charge, whereas the O and H atoms from the water molecules gain electrons, shifting to a more negative state. Consequently, the migration of O atoms from Region A to Region B signifies the continuous structural formation of Al oxide. Notably, under the applied electric fields of 200, 400, 600, and 800 V·m^−1^, the charge of the O atoms in Region B remains consistently within the range of −1.00 e to −1.25 e, confirming that the fundamental chemical valence of the newly formed Al oxide remains highly stable regardless of the external field strength.

**Figure 7:**
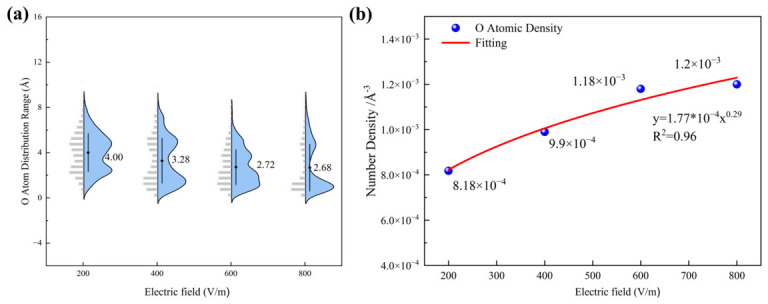
Quantitative analysis of the spatial growth of O atoms within Region B: (**a**) Distribution range of O atoms under different electric field strengths. (**b**) The atom density under different electric field strengths.

To further investigate the effect of electric field strength on the film formation, the distribution range of O atoms in the B region is statistically analyzed (Figure 6). The statistical range is the average value over 500 ps after the reaction reached equilibrium. From Figure 7a, it can be seen that as the electric field intensifies from 200 to 800 V·m^−1^, the average distribution range (thickness) of the O atoms noticeably narrows, measuring 4.00, 3.28, 2.72, and 2.68 Å, respectively. Furthermore, the O ion density under different electric field strengths was analyzed by combining it with the transformed number of O atoms shown in Figure 5. As illustrated in Figure 7b, the O atom densities are 8.18 × 10^−4^, 9.90 × 10^−4^, 1.18 × 10^−3^, and 1.20 × 10^−3^ atoms·Å^−3^ at 200, 400, 600, and 800 V·m^−1^, respectively. This systematic increase in O atoms per unit volume clearly demonstrates that a higher electric field strength promotes the formation of a denser oxide film.

### 3.2. Diffusion of H/O Atoms in the Al Substrate

**Figure 8:**
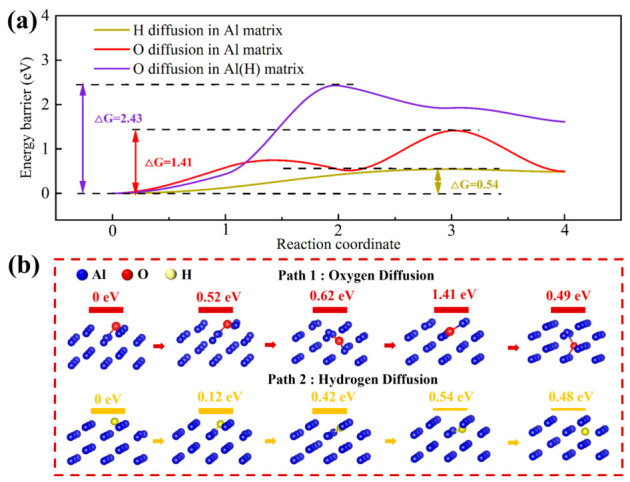
Diffusion behaviors of O and H atoms into the Al matrix: (**a**) Diffusion energy barrier of O and H, (**b**) Schematic diagram of H and O diffusion.

To clarify the sequence of formation and reaction mechanisms of oxyhydroxides and oxides, transition state calculations involving H and O were performed. It can be seen from Figure 8a that the energy barrier for H diffusion is 0.54 eV, whereas that for O reaches a substantial 1.41 eV. This profound disparity dictates that H atoms rapidly diffuse into the Al matrix prior to the O atoms. The interstitial diffusion sites are depicted in Figure 8b, which shows that H atoms preferentially occupy the octahedral sites, while O atoms reside in the tetrahedral sites of the Al lattice. Moreover, transition state calculations demonstrate that the pre-incorporation of H atoms effectively exerts a blocking effect, further elevating the O diffusion barrier to 2.43 eV. This synergistic hindrance strictly limits the subsequent inward penetration of O, conclusively demonstrating that Al oxyhydroxide preferentially forms and remains confined to the Al surface during the reaction.

To further evaluate the thermodynamic stability of O on the Al surface, adsorption energy calculations were performed. The adsorption of O on the H-containing Al (011) surface is energetically more favorable, with an adsorption energy of −0.0934 eV·Å^−2^, whereas the adsorption energy on the H-free surface is only −0.0879 eV·Å^−2^. The presence of H significantly enhances the adsorption affinity of O, which indicates that the H atoms in the water molecules can promote the formation of the oxide film after being incorporated into the Al matrix. Specifically, H atoms from water molecules can interact with O ions on the Al surface, thereby accelerating the formation of a hydrated oxide film.

**Figure 9:**
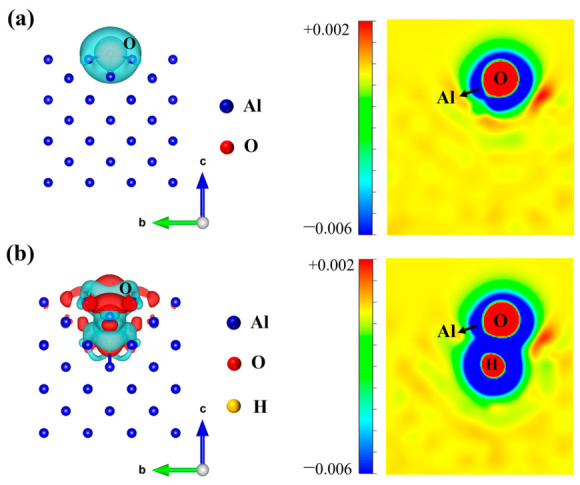
Charge density distribution of O atom adsorption. (**a**) Without H-doping, (**b**) With H-doping.

H atoms stably occupy the octahedral interstitial sites within the pure Al matrix. However, the subsequent adsorption of O atoms significantly redistributes the local electron cloud to form strong Al-O interactions. This local electronic perturbation alters the potential energy landscape around the H atom, driving its migration from the original octahedral site. To elucidate the electronic origin of this structural rearrangement, the charge density difference (CDD) was analyzed and visualized using the VESTA software (version 3.5.8) with an isosurface level of 0.0008 e/Å^3^ (Figure 9) [41]. Specifically, Figure 9a presents the CDD maps for the Al substrate without H atoms upon O adsorption. In comparison, Figure 9b shows the corresponding maps for the H-containing Al substrate. For the pure Al substrate, the CDD map reveals a localized charge rearrangement at the interface. Electron accumulation (red regions) is strictly localized around the O atom, surrounded by adjacent electron depletion (blue regions) on the topmost Al atoms. This confirms a conventional localized electron transfer from the top-layer Al substrate to the O atom, forming the initial Al-O bonds.

Conversely, the introduction of subsurface H atoms (Figure 9b) fundamentally alters the local electronic landscape. While the O atom still exhibits significant electron enrichment (indicated by the extensive red region), the most striking structural feature is the formation of a continuous and interconnected electron depletion region (blue zone) that physically bridges the surface O and the subsurface H atom. The surface O and subsurface H cooperatively withdraw electron density from the intervening Al atoms, demonstrating a strong long-range electronic coupling across the intermediate Al layers. This charge redistribution mechanism accounts well for the enhanced thermodynamic stability of O adsorption in the presence of subsurface H.

### 3.3. Effect of Multi-Step Anodization on Al Foil Tunnel Pores

**Figure 10:**
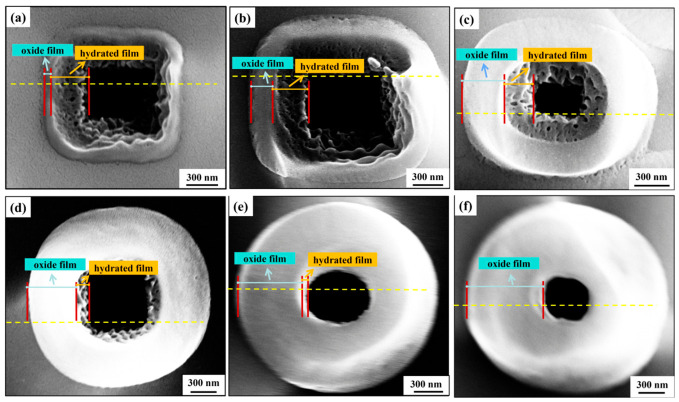
Oxide film on the surface of anodized foil pores: (**a**) Hydrated Film, (**b**) 200 V, (**c**) 360 V, (**d**) 520 V, (**e**) 680 V, (**f**) 800 V.

To investigate the effect of different anodization voltages on the film formation within the Al foil tunnel pores, the surface morphologies of the tunnel pores for the purely hydrated state and the samples anodized at 200, 360, 520, 680, and 800 V were examined, as displayed in Figure 10a–f respectively. It can be seen that the initial oxide film on the hydrated Al foil exhibits a wavy, loose, and highly porous structure, characteristic of the typical hydrated layer. Subsequently, multi-step voltages were applied to the Al foil. As the anodization voltage increased, the hydrated film gradually decreased. A new oxide film formed between the Al substrate and the hydrated film. Furthermore, the hydrated film became thinner, and the thickness of the new oxide film gradually increased. The quality of this oxide film plays a critical role in the energy storage capability of the anodized foil.

**Figure 11:**
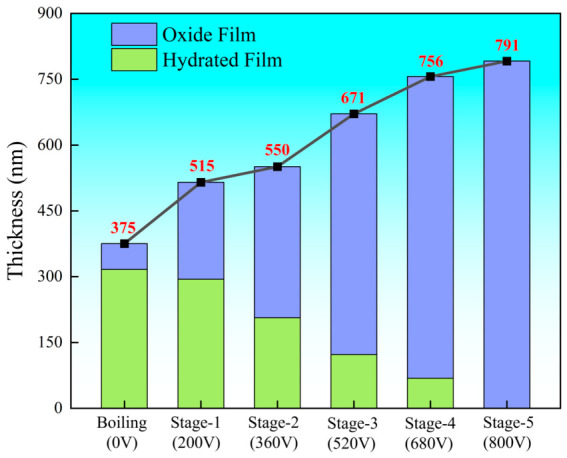
Variation in film thickness during the anodization process.

The film thicknesses were measured and presented in Figure 11. As the anodization voltage increases, the total thickness of the films significantly increases from an initial 375 nm to 791 nm. During anodization, the thickness of the hydrated film continuously decreased from the initial 316 nm and completely disappeared after the fifth step of anodization, while the oxide film formed and eventually grew to 791 nm. This phenomenon reveals two dominant mechanisms for film growth: firstly, the hydrated film undergoes dehydration, directly transforming into alumina, forming the foundation of the oxide film; secondly, under the influence of a strong electric field, Al atoms in the substrate are ionized and participate in the reaction, leading to the formation of new oxides, which further increase the thickness of the oxide film. Importantly, as the anodization voltage increases, the resulting pure oxide film exhibits enhanced compactness and a reduction in internal defects. This improved structural integrity effectively suppresses leakage current, which is highly beneficial for extending the service life of the ultra-high-voltage foil.

**Figure 12:**
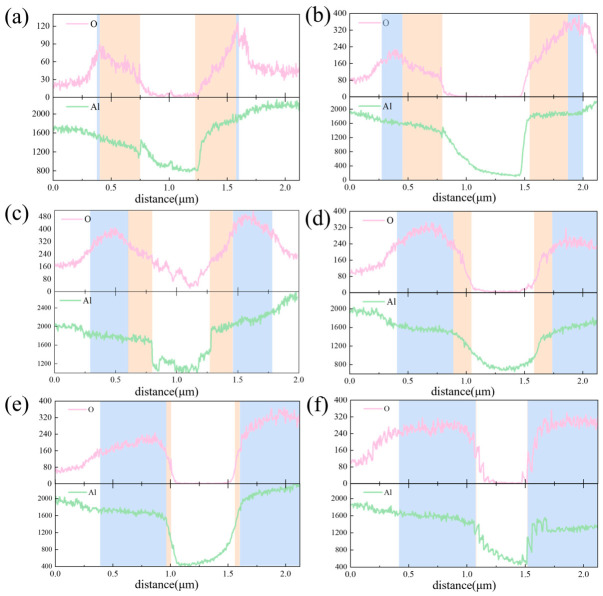
EDS line scan analysis of Al and O elements at different voltages: (**a**) Boiling; (**b**) 200 V; (**c**) 360 V; (**d**) 520 V; (**e**) 680 V; (**f**) 800 V.

Building upon the thickness measurements, EDS line scan analysis was conducted to further elucidate the interfacial compositional changes among the Al substrate, oxide film, and hydrated film. The results are shown in Figure 12. Along the scanning trajectory (yellow-dotted line), the fluctuation of the Al signal is minimal from the substrate to the tunnel pore. In contrast, the O signal shows a distinct trend, initially increasing and subsequently decreasing from the substrate toward the pore center. It is worth noting that a region with enhanced O signal intensity exists between the substrate and the hydrated film, confirming the formation of a uniform and dense alumina barrier layer at this interface. When scanning the transition from this dense oxide film outward to the hydrated film, a significant decrease in O concentration can be observed. In addition, with the increase in anodic oxidation voltage, the composition area of the dense Al_2_O_3_ barrier layer gradually widened. This indicates that the oxide layer is continuously thickened and densified under the continuous action of voltage. This experimental observation result is in good agreement with the early theoretical calculation (Figure 7), which demonstrated that higher electric field strengths essentially promote the formation of a denser oxide film.

**Figure 13:**
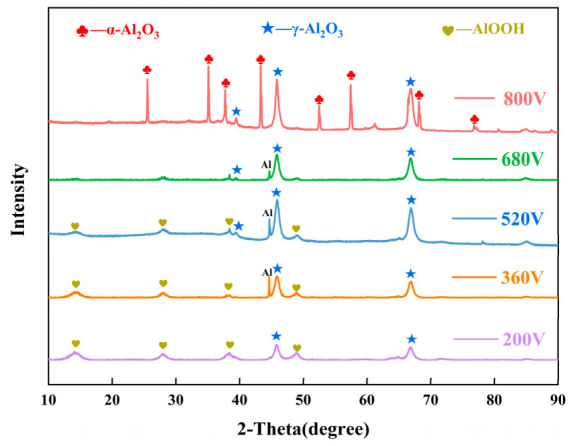
XRD patterns of the alumina films formed at different anodizing voltages.

While the preceding EDS analysis confirms that the anodic oxide layer primarily consists of Al and O elements, it cannot resolve the specific crystallographic structures. Therefore, XRD analysis was carried out to further identify the phase composition of the oxide film, as shown in Figure 13. The diffractograms display distinct characteristic peaks corresponding to boehmite (AlOOH), γ-Al_2_O_3_, and α-Al_2_O_3_. Notably, as the formation voltage increases, the phase composition undergoes a significant evolution. At lower voltages (200–520 V), the diffraction peaks of both AlOOH and γ-Al_2_O_3_ are clearly observed. Upon reaching 680 V, the film becomes predominantly composed of γ-Al_2_O_3_, and ultimately, at 800 V, α-Al_2_O_3_ emerges as the dominant phase.

### 3.4. Dielectric Properties of Al Oxide

As analyzed by the XRD, the hydroxide species (AlOOH) [35] initially formed on the Al surface undergo progressive, field-assisted dehydration during anodization, ultimately transforming into γ-Al_2_O_3_ and α-Al_2_O_3_ [36]. Since this structural evolution intrinsically dictates the macroscopic energy storage performance, DFT calculations systematically evaluate the dielectric properties of these key phases from an electronic perspective. Considering that γ-Al_2_O_3_ [42,43,44] exists in multiple structural configurations, several representative crystal models were selected for the calculations. The detailed crystallographic parameters and atomic coordinates of all structural models are provided in Section S4 of the Supporting Information.

Figure S1 (Supporting Information) illustrates the complex dielectric function spectra of six structural models: AlOOH, γ-Pinto, γ-Digne, γ-Ouyang, γ-Paglia, and α-Al_2_O_3_. The real and imaginary parts of the dielectric response represent the energy storage (polarization) and dielectric loss (energy absorption), respectively. The results reveal distinct dielectric behaviors across the different phases. In the low-frequency limit, the electronic contributions to the dielectric constant for the six models are 2.79, 1.91, 1.94, 1.23, 7.30, and 2.78, while the ionic contributions are 5.17, 5.32, 6.55, 5.33, 14.50, and 7.46, respectively. Within this low-frequency regime, the real part consistently exceeds the imaginary part for all models, indicating that the dielectric response is dominated by polarization-induced energy storage with negligible loss. As the frequency increases, the ionic and electronic polarizations exhibit distinct dispersion behaviors. The ionic response fluctuates significantly within its characteristic resonance region, where the imaginary part becomes dominant. Conversely, following high-frequency transitions, the electronic polarization stabilizes, with the real part remaining consistently larger than the imaginary part.

Typically, the dielectric constant of alumina is measured at low frequencies [45]. To accurately correlate with these experimental conditions, the static limit (*ω* = 0) is considered. At this zero-frequency limit, the total static dielectric constant of the Al oxide phases is determined by the sum of their corresponding electronic and ionic contributions.

**Figure 14:**
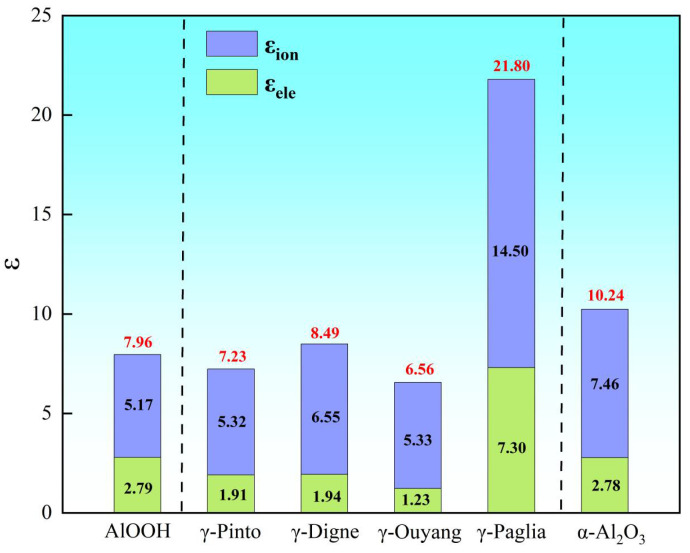
Dielectric constant of Al oxide at low frequencies.

The calculated low-frequency static dielectric constants for the various alumina phases are presented in Figure 14. As illustrated, significant variations exist among the different crystal structures. Specifically, the dielectric constant of AlOOH, which predominantly forms under low-voltage conditions, is 7.96. Among the different structural variants of γ-Al_2_O_3_, noticeable differences are also evident: the γ-Ouyang structure exhibits the lowest value (6.56), followed by the γ-Pinto (7.23) and γ-Digne (8.49) structures, while the γ-Paglia structure presents a remarkably high value of 21.80. Furthermore, the dense α-Al_2_O_3_ phase, which becomes dominant under high-voltage conditions, possesses a robust dielectric constant of 10.24. Crucially, this transition from the initial AlOOH to the highly crystalline α-Al_2_O_3_ explains the macroscopic experimental observation, confirming that the voltage-driven phase transformation intrinsically enhances the energy storage capability of the anodic foil. It should be noted that while the theoretical calculations verify that γ-Al_2_O_3_ exhibits a high intrinsic dielectric constant, which can significantly enhance the theoretical specific capacitance, actual device-level capacitance is also governed by complex macroscopic and engineering factors, which warrants further experimental exploration in future device applications.

These results demonstrate that the dielectric properties of alumina exhibit a pronounced dependence on the crystal structure, with substantial variations among different phases. Therefore, tailoring the anodizing conditions according to the dielectric characteristics of specific alumina phases is of great significance for enhancing the energy storage efficiency of Al electrolytic capacitors.

## 4. Conclusions

The effects of the applied electric field on the anodization dynamics and the dielectric properties of the resulting surface oxides were systematically investigated by combining experimental characterizations with ReaxFF-MD and DFT calculations. An 800 V ultra-high-voltage electrode foil was successfully prepared using a multi-step anodization process. The main conclusions are drawn as follows:

1. ReaxFF simulations indicate that the Al(011) plane exhibits the highest O reactivity, dominating the initial formation of the oxide film within the tunnel pores. Under an applied electric field, the alumina formed on this specific plane undergoes significant densification. Furthermore, DFT-based transition state calculations reveal that the diffusion barrier for hydrogen (0.54 eV) is substantially lower than that for O (1.41 eV). This kinetic advantage facilitates rapid hydrogen diffusion into the Al substrate, which synergistically promotes subsequent O adsorption. Consequently, in the early stages of anodization, Al hydroxides preferentially form on the pore surfaces prior to the direct generation of alumina.
2. During the multi-step anodization process, the total thickness of the films significantly increased from an initial 375 nm to 791 nm as the voltage increased. Concurrently, the initial 316 nm-thick hydrated layer was progressively consumed, being completely depleted after the fifth step of anodization. Driven by the strong electric field, this dehydration process induced a profound structural phase transformation, with the film composition systematically evolving from AlOOH to γ-Al_2_O_3_, and ultimately to highly crystalline α-Al_2_O_3_ at 800 V.
3. Calculations of dielectric properties show that AlOOH has a dielectric constant of 7.96, γ-Al_2_O_3_ (Paglia phase) exhibits the highest value of 21.80, and α-Al_2_O_3_ has a dielectric constant of 10.24. These results indicate that suppressing the formation of Al hydroxide during anodization and maximizing the content of high-dielectric-constant phases (such as γ-Al_2_O_3_ or α-Al_2_O_3_) is critical for enhancing the areal capacitance of Al electrolytic capacitors.

These findings provide both theoretical and experimental guidance for high-performance aluminum electrolytic capacitors. The 800 V ultra-high-voltage foil and the strategy to maximize high-dielectric-constant phases show great potential for energy storage in new energy vehicles and 5G communications. Future work will focus on optimizing anodization parameters to stabilize these optimal phases (e.g., γ-Al_2_O_3_) at scale, and further evaluating their long-term reliability and practical capacitance in actual devices.

## Figures and Tables

**Table 1:** Thinning time for anodized foil under different anodization processes.

Process	Boiling	Anodization Step				
		1st	2nd	3rd	4th	5th
Voltage (V)	0	200	360	520	680	800
Time (min)	60	90	120	180	240	300

## Data Availability

The original contributions presented in this study are included in the article/Supplementary Material. Further inquiries can be directed to the corresponding authors.

## Supplementary Materials

The following supporting information can be downloaded at: https://www.mdpi.com/article/10.3390/ma19112373/s1.
