# Supplementary material for: Formation Mechanism and Dielectric Properties of Ultra-High-Voltage Anodic Al Foils Investigated by ReaxFF-MD and DFT
Source: Materials (Basel). 2026 Jun 3;19(11):2373. doi: 10.3390/ma19112373 (PMC13258237; doi:10.3390/ma19112373)
Supplement: Supplementary file 1 [file materials-19-02373-s001.zip › materials-4295755-supplementary.pdf]

S1. ReaxFF Energy Function Description

The total potential energy of the system within the Reactive Force Field (ReaxFF) framework is expressed as follows:

$$E_{system} = E_{bond} + E_{over} + E_{under} + E_{vals} + E_{tor} + E_{lp} + E_{H-bonds} + E_{valwads} + E_{coulomb} \quad \text{(Eq.S1)}$$

where E_{bonds}, E_{lp}, E_{over}, and E_{under} represent bond, lone pair, over-coordination, and under-coordination energies, respectively. E_{tor} and $E_{H\text{-}bonds}$ correspond to the torsional angle energy and H-bond energy. $E_{valwads}$ and $E_{coulomb}$ represent non-bonding energies, i.e., van der Waals and Coulomb forces, which are calculated for all atomic pairs.

S2. Surface Adsorption Energy Formulation

To evaluate the thermodynamic stability of the adsorbed species on the aluminum surfaces, the adsorption energy (E_{ads}) is defined by the following equation:

$$E_{\mathrm{ads}} = E_{surface+O} - (E_{surface} + E_O) \quad \text{(Eq.S2)}$$

where $E_{surface+O}$ is the total energy of the system after the O atom is adsorbed; $E_{surface}$ is the energy of the Al surface; and E_O is the energy of a single O atom.

S3. Static Dielectric Constant Calculation

The frequency-dependent total static dielectric constant is partitioned into electronic and ionic (lattice) polarization contributions, as expressed below:

$$\varepsilon_{(\omega)} = \varepsilon_{\mathrm{ele}(\omega)} + \varepsilon_{\mathrm{ion}(\omega)} \quad \text{(Eq.S3)}$$

where $\varepsilon_{(\omega)}$ represents the total dielectric constant at frequency ω, while $\varepsilon_{ele(\omega)}$ and $\varepsilon_{ion(\omega)}$ denote the contributions from electronic polarization and ionic polarization, respectively.

S4. Crystallographic Coordinates of the Structural Models

To ensure the reproducibility of the first-principles calculations, the optimized crystallographic structures of the various γ-Al2O3 configurations discussed in the main text are provided here in the POSCAR format. These files contain the precise lattice vectors and atomic coordinates used as the direct input for our DFT simulations. Each model represents a distinct structural arrangement within the γ-Al2O3 landscape as referenced in the manuscript.

γ-Al_2O_3(Ouyang)

```
   1.0
   7.8582886538942764    -0.0014507897449220     0.0000000000000000
   -0.0014489126276008    7.8462212453022886     0.0000000000000000
   0.0000000000000000     0.0000000000000000    23.5504430759552861
   O      Al
   96     64
Direct
  0.3883376688668101   0.1057639359123934   0.0457207306981475
  0.1371596823227778   0.3800890055390471   0.0389523536586014
```

0.8859751673056520	0.6385913762991370	0.0393753396341941
0.6113756484023298	0.8739477698760368	0.0387407706994199
0.8579685809655332	0.1012972869954801	0.0459851021568401
0.6108511081160887	0.3643751124772734	0.0444710844618317
0.3614694322282592	0.6339981504002582	0.0458894015658969
0.1314023130728959	0.8644733908216315	0.0447108427464918
0.6338251503239968	0.1291241847238653	0.1229455073030714
0.8622234645125059	0.3648023998111129	0.1253947059961434
0.1290615382090691	0.6324489831043978	0.1234398393626562
0.3635501539332359	0.8646553795867643	0.1216011340967508
0.1198110932643291	0.1177610024225998	0.1265207497208568
0.3862551987003296	0.3857979497574337	0.1245670981445262
0.6142908887530947	0.6210702736257537	0.1270273769216641
0.8849492307878123	0.8849920568277920	0.1284611009416004
0.8873535836024995	0.1161692396561768	0.2035658128049395
0.6364705801874012	0.3855452117983509	0.2049095354338076
0.3900547027071929	0.6494240035618197	0.2045743984072388
0.1172869761312859	0.8855770050253936	0.2057972145615367
0.3637246295499039	0.1129173634569308	0.2104492193342061
0.1124484509741894	0.3710206800107002	0.2109015470630298
0.8594368594095976	0.6437461653435180	0.2042734110850412
0.6363730684970664	0.8756858511332791	0.2113245347330413
0.1388977169790117	0.1398978020685694	0.2874787333672189
0.3630150957643982	0.3699914752725825	0.2971785239277147
0.6101352402520969	0.6441797190460659	0.2898087564581153
0.8622288365721774	0.9014377816819859	0.2881692272365903
0.6171574668539719	0.1286854992431154	0.2939953981789175
0.8899068356747450	0.3648724136019242	0.2955955417505968
0.1414033769553860	0.6268897983503421	0.2959239897060437
0.3879838119378913	0.8990616760488449	0.2960873665426275
0.3848214084085361	0.1292466122444599	0.3737466546833705
0.1141512741593898	0.3911882318683766	0.3709421862677020
0.8842071477584891	0.6261065574569967	0.3790366221499653
0.6191584277038167	0.8964314190898691	0.3717831791748558
0.8677743772745723	0.1426221341929563	0.3780249582367522
0.6338827306364796	0.3758346925346329	0.3767094029101050
0.3659484847090913	0.6482259929234978	0.3790159420558452
0.1350180973983758	0.8812891403014128	0.3709726190652486
0.6357249544578432	0.1472024863959190	0.4540521280436577
0.8672911945808487	0.3741227653895052	0.4561654102634401
0.1430996494822915	0.6144303298793391	0.4531340749939494
0.3919069505176506	0.8795580097476372	0.4552683845387822
0.1168570740789044	0.1501181564210285	0.4610054964250292
0.3900081989746674	0.3866959249764955	0.4614936453295713

0.6101358616016761	0.6200675639047916	0.4542469166942667
0.8597324776826014	0.8971676856090585	0.4609696486908055
0.8886243515976702	0.1260522301239603	0.5387407706994246
0.6140248326943480	0.3614086237008610	0.5393753396341940
0.3628403176772232	0.6199109944609534	0.5389523536586036
0.1116623311331908	0.8942360640876074	0.5457207306981458
0.3685976869271041	0.1355266091783674	0.5447108427464890
0.1385305677717397	0.3660018495997409	0.5458894015658970
0.8891488918839113	0.6356248875227271	0.5444710844618333
0.6420314190344668	0.8987027130045185	0.5459851021568394
0.1364498460667645	0.1353446204132340	0.6216011340967514
0.3709384617909337	0.3675510168956015	0.6234398393626536
0.6377765354874941	0.6351976001888889	0.6253947059961437
0.8661748496760032	0.8708758152761362	0.6229455073030711
0.6150507692121877	0.1150079431722115	0.6284611009416007
0.8857091112469053	0.3789297263742495	0.6270273769216692
0.1137448012996710	0.6142020502425652	0.6245670981445267
0.3801889067356703	0.8822389975773984	0.6265207497208561
0.3827130238687128	0.1144229949746038	0.7057972145615342
0.1099452972928071	0.3505759964381792	0.7045743984072383
0.8635294198125988	0.6144547882016480	0.7049095354338072
0.6126464163975005	0.8838307603438214	0.7035658128049407
0.8636269315029336	0.1243141488667250	0.7113245347330456
0.6405631405904024	0.3562538346564851	0.7042734110850437
0.3875515490258129	0.6289793199893017	0.7109015470630281
0.1362753704500951	0.8870826365430696	0.7104492193342042
0.6377711634278226	0.0985622183180111	0.7881692272365873
0.8898647597479031	0.3558202809539337	0.7898087564581115
0.1369849042356025	0.6300085247274175	0.7971785239277136
0.3611022830209885	0.8601021979314314	0.7874787333672197
0.1120161880621075	0.1009383239511543	0.7960873665426293
0.3585966230446159	0.3731102016496602	0.7959239897060461
0.6100931643252550	0.6351275863980742	0.7955955417505959
0.8828425331460281	0.8713145007568841	0.7939953981789168
0.8808415722961833	0.1035685809101328	0.8717831791748560
0.6157928522415109	0.3738934425430050	0.8790366221499654
0.3858487258406106	0.6088117681316279	0.8709421862677040
0.1151785915914640	0.8707533877555388	0.8737466546833723
0.3649819026016244	0.1187108596985873	0.8709726190652468
0.1340515152909108	0.3517740070765003	0.8790159420558445
0.8661172693635204	0.6241653074653631	0.8767094029101068
0.6322256227254277	0.8573778658070420	0.8780249582367511
0.1080930494823491	0.1204419902523645	0.9552683845387839
0.3569003505177099	0.3855696701206627	0.9531340749939495

0.6327088054191513	0.6258772346104906	0.9561654102634402
0.8642750455421568	0.8527975136040811	0.9540521280436565
0.6402675223173986	0.1028323143909419	0.9609696486908038
0.8898641383983239	0.3799324360952034	0.9542469166942689
0.1099918010253331	0.6133040750235029	0.9614936453295729
0.3831429259210942	0.8498818435789715	0.9610054964250286
-0.0102545975961837	-0.0098057568345208	-0.0000345417725555
0.4901475998520824	0.4946986507737103	-0.0000828627387403
0.6229957870814840	0.1343276798663681	0.0373262011127068
0.1169413589599420	0.6100859368186826	0.0429137746177971
0.3701509434653677	0.8776354745212988	0.0384175130363180
0.2615459531462533	0.2508897960164764	0.0826791166950193
0.7498760941598339	0.7515107106436869	0.0839280313177899
0.8760901720393512	0.1353273037442078	0.1214035360573079
0.6240678914472928	0.3753689652559060	0.1246996997155148
0.3723144723529527	0.6151256719717108	0.1288077577106343
0.1244486851787770	0.8748709738705926	0.1252331220740312
0.4983365097675541	-0.0019428978979507	0.1659915069773750
-0.0125769225814401	0.4998178129881187	0.1672225279544951
0.1326970730514137	0.1409125423735841	0.2067655930222074
0.6246754927539293	0.6149226755060634	0.2128646304311075
0.8777891385603629	0.8718531475222574	0.2113515074789037
0.7587204812384628	0.2552696947591738	0.2484714766832208
0.2584013402151879	0.7596262161275324	0.2515785232255863
0.3796828846539501	0.1430257766349144	0.2875381496410744
0.1268203051483756	0.3979351935256846	0.2879724715264925
0.6318427275040993	0.8747422193153737	0.2927697285056607
0.0013569743096963	0.0134680768418279	0.3319588620772258
0.4928199025456904	0.5116850047283418	0.3343118946440334
0.6261660566432427	0.1369262124958767	0.3747552404396999
0.8739938277801956	0.3963528540275733	0.3714354649132167
0.1152101325371889	0.6481269825057603	0.3753627509803100
0.3651577490479028	0.8903273004008614	0.3783110467660882
0.2492733529405765	0.2678216202104850	0.4163094450184728
0.7465073367988545	0.7570412124745323	0.4176991393551894
0.8854796690077228	0.1270185933760580	0.4572790619483713
0.6315920156527393	0.3935450746940279	0.4623460926632597
0.3752210879432262	0.6470727871814178	0.4620099196994818
0.5102545975961847	0.0098057568345208	0.4999654582274448
0.0098524001479188	0.5053013492262873	0.4999171372612585
0.1298490565346322	0.1223645254787039	0.5384175130363231
0.3830586410400604	0.3899140631813181	0.5429137746177969
0.8770042129185160	0.8656723201336302	0.5373262011127073
0.7501239058401661	0.2484892893563112	0.5839280313177951

0.2384540468537462	0.7491102039835242	0.5826791166950227
0.3755513148212222	0.1251290261294081	0.6252331220740301
0.1276855276470494	0.3848743280282890	0.6288077577106359
0.8759321085527072	0.6246310347440943	0.6246996997155160
0.6239098279606488	0.8646726962557915	0.6214035360573081
0.0016634902324472	0.0019428978979507	0.6659915069773753
0.5125769225814368	0.5001821870118822	0.6672225279544971
0.6222108614396371	0.1281468524777400	0.7113515074789064
0.8753245072460707	0.3850773244939361	0.7128646304311090
0.3673029269485855	0.8590874576264170	0.7067655930222071
0.2415986597848127	0.2403737838724659	0.7515785232255864
0.7412795187615372	0.7447303052408257	0.7484714766832226
0.8681572724959007	0.1252577806846252	0.7927697285056622
0.3731796948516247	0.6020648064743112	0.7879724715264944
0.1203171153460496	0.8569742233650878	0.7875381496410776
0.4986430256903036	-0.0134680768418279	0.8319588620772238
0.0071800974543096	0.4883149952716578	0.8343118946440327
0.1348422509520978	0.1096726995991403	0.8783110467660868
0.3847898674628089	0.3518730174942417	0.8753627509803089
0.6260061722198044	0.6036471459724273	0.8714354649132137
0.8738339433567573	0.8630737875041243	0.8747552404396983
0.7534926632011455	0.2429587875254661	0.9176991393551889
0.2507266470594226	0.7321783797895175	0.9163094450184742
0.1247789120567746	0.3529272128185828	0.9620099196994810
0.8684079843472607	0.6064549253059721	0.9623460926632579
0.6145203309922772	0.8729814066239431	0.9572790619483754

γ-Al_2O_3(Digne)

1.0

5.4772525727018753	0.0000000000000000	-0.0014946053593966
0.0000000000000000	8.2551054796091154	0.0000000000000000
-0.0836667600380785	0.0000000000000000	7.9630433369267779

Al O

16 24

Direct

0.3783617630920590	0.7500000000000000	0.1256268180697147
0.6216382669079421	0.2500000000000000	0.8743731969302821
0.8683244316272903	0.2500000000000000	0.4993431296490661
0.1316755683727093	0.7500000000000000	0.5006568403509307
0.8762339481933428	0.7500000000000000	0.1251855120967461
0.1237660518066577	0.2500000000000000	0.8748144879032543
0.6165188037424932	0.7500000000000000	0.7464246207839950

0.3834811962575070	0.2500000000000000	0.2535753792160043
0.3674998129293041	0.0756905985568402	0.6133159282348807
0.6325002170706922	0.9243094164431580	0.3866840717651198
0.6325002170706922	0.5756905835568420	0.3866840717651198
0.3674998129293041	0.4243094164431569	0.6133159282348807
0.1174230360074153	0.5782941095233939	0.8628782607614646
0.8825769639925850	0.4217058904766081	0.1371217392385349
0.8825769639925850	0.0782941095233922	0.1371217392385349
0.1174230360074153	0.9217058904766061	0.8628782607614646
0.8827894425060588	0.7500000000000000	0.8777791198238264
0.1172105574939423	0.2500000000000000	0.1222208801761745
0.6115193941459263	0.2500000000000000	0.6402182322761679
0.3884806058540727	0.7500000000000000	0.3597817677238319
0.3617402876252415	0.7500000000000000	0.8894222936271482
0.6382597423747601	0.2500000000000000	0.1105777063728509
0.1337584424039909	0.2500000000000000	0.6291134397257246
0.8662415875960112	0.7500000000000000	0.3708865602742741
0.8861916500613656	0.4065216587288715	0.8977570139463973
0.1138083499386344	0.5934783112711255	0.1022429860536019
0.1138083499386344	0.9065216887288745	0.1022429860536019
0.8861916500613656	0.0934783412711286	0.8977570139463973
0.6070346972903577	0.9182713150811079	0.6138250755797514
0.3929653027096441	0.0817286849188933	0.3861749244202476
0.3929653027096441	0.4182713150811064	0.3861749244202476
0.6070346972903577	0.5817286849188921	0.6138250755797514
0.3582929204651505	0.4066639741343141	0.8547279124202578
0.6417070795348508	0.5933359958656834	0.1452720875797421
0.6417070795348508	0.9066640041343166	0.1452720875797421
0.3582929204651505	0.0933360258656863	0.8547279124202578
0.1345859596139900	0.9174140900433226	0.6369964455789705
0.8654140263860102	0.0825859099566766	0.3630035544210281
0.8654140263860102	0.4174140900433242	0.3630035544210281
0.1345859596139900	0.5825859099566774	0.6369964455789705

γ-Al_2O_3(Pinto)

1.0

5.5582494006399186 -0.0008974858205014 0.0568904544709482

2.7445872844688148 4.8333609262054669 0.0568904544711849

-0.0029444337396718 -0.0017137358495684 13.4558307650925570

Al O

16 24

Direct

-0.0021562345225163	-0.0021562345225163	0.1221284211703340
1.0021562425225243	1.0021562425225243	0.8778715788296667
0.6761276413367742	0.6761276413367742	0.2076238666573468
0.3238723586632256	0.3238723586632256	0.7923761183426588
0.3328330066294330	0.3328330066294330	0.5467522778088958
0.6671669633705601	0.6671669633705601	0.4532477221911082
0.0000000000000000	0.5000000000000000	0.0000000000000000
0.5000000000000000	0.0000000000000000	0.0000000000000000
0.3481651063342316	0.3481651063342316	0.1603217380517533
0.6518349226657644	0.6518349226657644	0.8396782479482456
0.8331890359504013	0.8331890359504013	0.6609230784032969
0.1668109640495965	0.1668109640495965	0.3390769215967047
0.6765550115196652	0.1660535310354657	0.3397704048290337
0.3234449884803334	0.8339464539645365	0.6602295661709696
0.1660535310354657	0.6765550115196652	0.3397704048290337
0.8339464539645365	0.3234449884803334	0.6602295661709696
0.1556527527485489	0.6539706554435963	0.0856591213911829
0.8443472172514600	0.3460293445564078	0.9143408706088139
0.1625774618917529	0.6679969129046109	0.5935517948253116
0.8374225081082440	0.3320030870953853	0.4064482051746877
0.5105089944816732	0.9671784463212123	0.7456308180880651
0.4894910055183245	0.0328215536787855	0.2543691819119338
0.1777991938855742	0.1777991938855742	0.0837659061210488
0.8222008061144245	0.8222008061144245	0.9162340868789434
0.3214342337185854	0.3214342337185854	0.9210381304928578
0.6785657962814071	0.6785657962814071	0.0789618695071440
0.1633778943199689	0.1633778943199689	0.5937088037774921
0.8366221056800338	0.8366221056800338	0.4062911962225017
0.5051935757360811	0.5051935757360811	0.7472741700837289
0.4948064242639165	0.4948064242639165	0.2527258299162719
0.9957944973611937	0.9957944973611937	0.7419404594873866
0.0042055026388076	0.0042055026388076	0.2580595405126143
0.6636774577831169	0.6636774577831169	0.5877544985727985
0.3363225422168814	0.3363225422168814	0.4122455014272021
0.9671784463212123	0.5105089944816732	0.7456308180880651
0.0328215536787855	0.4894910055183245	0.2543691819119338

0.6679969129046109	0.1625774618917529	0.5935517948253116
0.3320030870953853	0.8374225081082440	0.4064482051746877
0.6539706554435963	0.1556527527485489	0.0856591213911829
0.3460293445564078	0.8443472172514600	0.9143408706088139

γ-Al_2O_3(Paglia)

1.0

2.9204722221082515 -0.0583306749880538 -0.4621945088207259

0.2630077151962512 5.2200672630237213 0.3229810764332738

-2.1139942050546479 -0.8034536679458735 6.6791019150616382

Al O

4 6

Direct

0.3658644825330652 0.5271177632558519 -0.8384209422081378

0.3418723947870213 0.8478005954935621 0.4978143131332146

1.0002625469335513 0.3595688537371912 1.6571255080806990

-1.1194618520653510 0.0817044440601278 0.0205733262445453

2.2560370127062308 1.1905690375949165 0.9010865093116882

0.0967837585906482 0.8432874222036704 0.2067350098635251

0.8287657460029352 0.6357679969781362 0.4795919056331170

0.3873849523057895 0.1895389414389247 1.5497036822207577

0.6392632894023310 0.8222789776633881 -0.2091421844296438

-0.1534722023706183 0.5709662399705169 0.8466320296360786

S5. Electronic and Ionic Contributions to the Dielectric Response

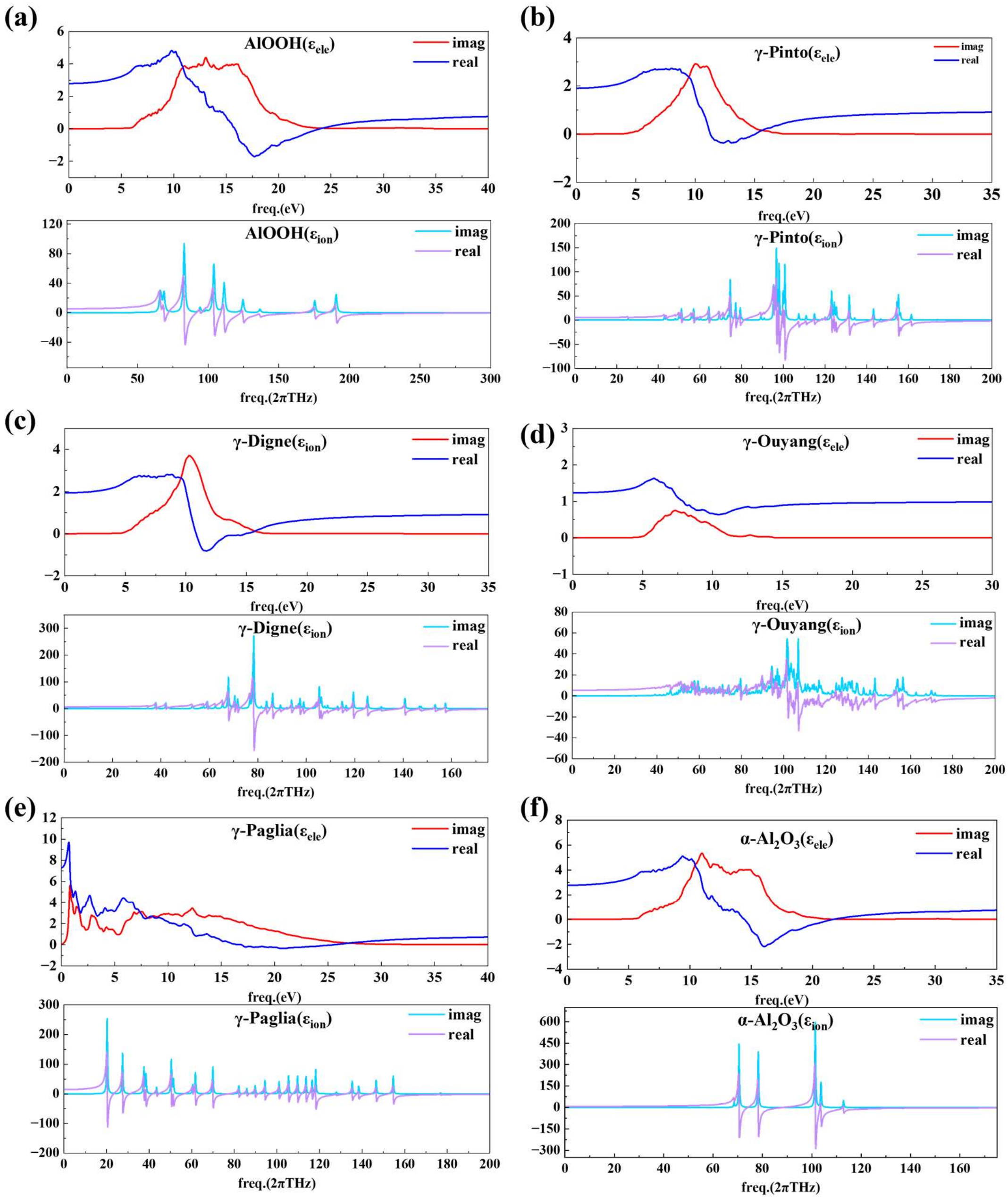

Figure S1. Dielectric Function Contributions of Al Oxide. Electronic and Ionic Contributions: (a) AlOOH; (b-e) γ-Al_2O_3; (f) α-Al_2O_3.
